# ACSL4 as a context-dependent metabolic switch in hepatocellular carcinoma: implications for ferroptosis and immunotherapy

**DOI:** 10.3389/fimmu.2026.1821702

**Published:** 2026-04-15

**Authors:** Qianbin Luo, Zhengli Zhang

**Affiliations:** Department of Gastroenterology, Shuguang Hospital Affiliated to Shanghai University of Traditional Chinese Medicine, Shanghai, China

**Keywords:** ACSL4, ferroptosis, hepatocellular carcinoma, lipid metabolic reprogramming, tumor immunity

## Abstract

Hepatocellular carcinoma (HCC) is characterized by profound lipid metabolic rewiring that supports tumor growth, therapeutic resistance, and immune evasion. Among lipid metabolic regulators, acyl-CoA synthetase long-chain family member 4 (ACSL4) has emerged as a pivotal determinant of polyunsaturated fatty acid (PUFA) activation and membrane phospholipid remodeling. Accumulating evidence reveals a functional duality of ACSL4 in HCC. On one hand, ACSL4 amplifies lipogenic transcriptional programs, enhances fatty acid oxidation–mediated energy adaptation, and cooperates with oncogenic signaling networks to promote tumor proliferation and survival, particularly under nutrient stress such as transarterial chemoembolization (TACE). On the other hand, ACSL4-driven enrichment of PUFA-containing phospholipids establishes the biochemical foundation for ferroptosis, sensitizing tumor cells to sorafenib and CD8^+^ T cell–mediated oxidative killing. This apparent paradox can be reconciled by conceptualizing ACSL4 as a context-dependent metabolic switch. Its biological output is dynamically tuned by therapeutic modality, microenvironmental redox conditions, post-transcriptional regulation (e.g., miR-23a-3p and miR-145-5p), post-translational modification (e.g., SIAH2-mediated ubiquitination), and substrate flux partitioning. Through these multilayered regulatory mechanisms, ACSL4 integrates lipid remodeling with ferroptotic sensitivity and tumor–immune interactions within the tumor microenvironment. In this mini-review, we synthesize recent mechanistic and translational findings to propose a unifying framework for ACSL4 function in HCC. Understanding ACSL4 as a metabolic switch rather than a static oncogenic factor may enable rational design of ferroptosis-enhancing and immunometabolic therapeutic strategies and support biomarker-guided precision medicine in HCC.

## Highlights

- ACSL4 integrates lipid remodeling with ferroptotic sensitivity in hepatocellular carcinoma.
- ACSL4 functions as a context-dependent metabolic switch, promoting tumor growth or ferroptosis depending on therapeutic and microenvironmental cues.
- Targeting ACSL4 regulatory networks may enhance ferroptosis-based therapy and immunotherapy in HCC.

## 1 Introduction

Hepatocellular carcinoma (HCC) is a prototypical metabolically reprogrammed malignancy arising from a physiologically lipid-rich organ (1). Unlike many other solid tumors that predominantly rely on glycolytic reprogramming, HCC exhibits profound alterations in lipid metabolism, reflecting both the intrinsic metabolic functions of hepatocytes and the adaptive demands of tumor progression (2, 3). *De novo* lipogenesis is frequently upregulated in HCC, driven by transcriptional programs such as c-Myc and SREBP1, resulting in enhanced synthesis of triglycerides, cholesterol, and membrane phospholipids (4). Concurrently, aberrant arachidonic acid metabolism and remodeling of PUFA pools contribute to membrane composition changes and redox vulnerability. In addition to lipid synthesis, fatty acid β-oxidation (FAO) is dynamically regulated in HCC (5). Under conditions of metabolic stress—such as glucose deprivation, hypoxia, or therapeutic embolization—tumor cells increasingly rely on lipid-derived substrates to sustain mitochondrial ATP production. This metabolic flexibility enables HCC cells to survive fluctuating nutrient availability and therapeutic pressure. Importantly, emerging evidence suggests that such lipid metabolic plasticity is not merely a byproduct of tumor growth but a determinant of therapeutic responsiveness, influencing outcomes following transarterial chemoembolization (TACE), targeted therapy, and immunotherapy. Thus, lipid metabolic rewiring in HCC represents a dynamic and context-dependent process that integrates anabolic growth signals with stress-adaptive energy programs.

Ferroptosis is an iron-dependent, lipid peroxidation–driven form of regulated cell death that has emerged as a critical vulnerability in HCC (6). Mechanistically, ferroptosis depends on the accumulation of oxidizable PUFA-containing phospholipids (PUFA-PLs) within cellular membranes (7, 8). The availability and incorporation of these substrates are tightly regulated by lipid metabolic enzymes, most notably acyl-CoA synthetase long-chain family member 4 (ACSL4). By catalyzing the activation of long-chain PUFAs and facilitating their esterification into membrane phospholipids, ACSL4 determines the size and composition of the ferroptosis-sensitive lipid pool (9, 10). In HCC, ferroptosis has gained considerable attention as a therapeutic target, particularly in the context of sorafenib-based treatment, where induction of ferroptotic stress contributes to anti-tumor efficacy. However, ferroptotic sensitivity is highly heterogeneous and dynamically regulated. Tumor cells can modulate ferroptosis thresholds through transcriptional, epigenetic, and post-translational mechanisms, thereby influencing drug resistance. Beyond tumor cell–intrinsic effects, ferroptosis is increasingly recognized as an immunologically relevant process. Lipid peroxidation products, metabolic byproducts, and ferroptotic stress signals shape the tumor microenvironment (TME), affecting macrophage polarization, T-cell function, and immune checkpoint responsiveness. Conversely, immune effector cells such as CD8^+^ T cells can promote ferroptosis in tumor cells by altering cystine metabolism and redox balance. Therefore, ferroptosis lies at the intersection of tumor metabolism and anti-tumor immunity, making it a central node in immunometabolic regulation.

ACSL4 occupies a unique position at the crossroads of lipid metabolism and ferroptosis. As a PUFA-activating enzyme, ACSL4 catalyzes the conversion of long-chain PUFAs—particularly arachidonic acid and adrenic acid—into their corresponding acyl-CoA derivatives, enabling their incorporation into membrane phospholipids (11, 12). In doing so, ACSL4 directly determines the availability of ferroptotic substrates and thus the intrinsic susceptibility of tumor cells to lipid peroxidation–induced death (13). Paradoxically, the same enzymatic activity that sensitizes cells to ferroptosis can also promote tumor growth. By fueling lipid remodeling, supporting membrane biogenesis, and facilitating adaptive fatty acid oxidation under nutrient stress, ACSL4 contributes to metabolic fitness and tumor progression (14–16). In HCC, ACSL4 has been implicated in *de novo* lipogenesis, transcriptional signaling networks, mitochondrial energy adaptation, and immune modulation, underscoring its multifaceted roles (17, 18). This apparent functional duality suggests that ACSL4 does not act as a simple oncogenic driver or tumor suppressor. Instead, its biological output depends on metabolic context, therapeutic pressure, and microenvironmental cues. ACSL4 may therefore function as a context-dependent metabolic switch, dynamically balancing lipid remodeling, ferroptotic sensitivity, and tumor–immune interactions.

In this review, we propose a unifying framework in which ACSL4 functions as a context-dependent metabolic switch that integrates lipid remodeling, ferroptosis sensitivity, and tumor–immune interactions in hepatocellular carcinoma. Unlike conventional discussions that describe ACSL4 primarily as either a ferroptosis executor or a metabolic enzyme, we emphasize its dynamic and bidirectional roles under distinct therapeutic and microenvironmental conditions. By synthesizing recent mechanistic, immunological, and translational evidence, this review provides a conceptually integrated perspective on how ACSL4-driven lipid plasticity shapes treatment response, biomarker stratification, and rational combination strategies in HCC.

## 2 ACSL4 in lipid metabolic reprogramming: pro-tumorigenic functions

### 2.1 ACSL4–c-Myc–SREBP1 axis and *De Novo* lipogenesis

*De novo* lipogenesis is a hallmark of hepatocellular carcinoma and serves as a critical driver of membrane biogenesis, oncogenic signaling, and metabolic buffering (19, 20). Among the lipid metabolic enzymes implicated in this process, ACSL4 has emerged as an upstream regulator of lipogenic transcriptional programs. Beyond its canonical role in activating long-chain PUFAs, ACSL4 contributes to the amplification of anabolic lipid synthesis through the c-Myc–SREBP1 axis (17). Mechanistically, ACSL4 upregulation enhances c-Myc activity, which in turn promotes the transcriptional activation of sterol regulatory element-binding protein 1 (SREBP1), a master regulator of fatty acid and cholesterol biosynthesis. Activated SREBP1 drives the expression of downstream lipogenic enzymes, resulting in increased accumulation of triglycerides, cholesterol, and lipid droplets within tumor cells. This lipid enrichment supports rapid membrane expansion, organelle biogenesis, and the assembly of signaling platforms required for sustained proliferation. Functionally, enforced ACSL4 expression enhances HCC cell growth, migration, and invasion, whereas genetic silencing attenuates lipogenic flux and suppresses tumor progression *in vitro* and *in vivo*. Notably, restoration of SREBP1 can partially rescue the anti-proliferative effects induced by ACSL4 inhibition, underscoring the centrality of the ACSL4–c-Myc–SREBP1 cascade in driving lipogenesis-dependent tumor growth. These findings position ACSL4 not merely as a lipid-activating enzyme but as a metabolic amplifier of oncogenic transcriptional circuitry.

### 2.2 ACSL4 in fatty acid oxidation and energy adaptation

While ACSL4 promotes lipid synthesis under nutrient-replete conditions, its role extends to facilitating energy adaptation under metabolic stress. HCC frequently develops in a fibrotic and hypoxic microenvironment characterized by fluctuating glucose availability. Therapeutic interventions such as TACE further impose ischemic and nutrient-deprived conditions, forcing tumor cells to rewire their metabolic dependencies. Under glucose starvation, ACSL4-mediated activation of arachidonic acid (AA) provides substrates for mitochondrial β-oxidation, thereby sustaining ATP production. This shift toward lipid-derived energy sources enables tumor cells to survive glucose limitation and maintain redox homeostasis. Importantly, suppression of ACSL4 under these stress conditions impairs mitochondrial respiration and compromises tumor cell viability, highlighting its role in metabolic resilience. Recent evidence further implicates ACSL4 within a broader regulatory network involving RNA-binding proteins and mTOR signaling. The RBM45–Rictor–ACSL4 axis represents a metabolic coordination platform in which RBM45 enhances lipid metabolic remodeling and interacts with Rictor (a core component of mTORC2), promoting both fatty acid synthesis and oxidation pathways (21). This dual regulation underscores the capacity of ACSL4 to integrate anabolic and catabolic lipid fluxes, thereby increasing metabolic flexibility. Such adaptability is particularly advantageous in the heterogeneous and stress-prone tumor microenvironment of HCC.

### 2.3 Interaction with transcriptional programs: ACSL4 as a signaling node

Beyond metabolic flux control, ACSL4 has been linked to transcriptional and signaling programs that further reinforce malignant phenotypes. Notably, ACSL4 can influence the transcriptional activation of p21-activated kinase 2 (PAK2), a kinase involved in cytoskeletal remodeling, cell motility, and survival signaling (22).

Evidence suggests that ACSL4 enhances PAK2 expression through modulation of the transcription factor Sp1. This ACSL4–Sp1–PAK2 axis establishes a feed-forward loop in which lipid metabolic remodeling is coupled to pro-migratory and pro-survival signaling pathways (22). As a result, ACSL4 contributes not only to metabolic reprogramming but also to the activation of downstream oncogenic circuits that facilitate tumor invasion and metastasis. These findings challenge the traditional view of metabolic enzymes as passive executors of biochemical reactions. Instead, ACSL4 appears to function as a metabolic–transcriptional hub, bridging lipid metabolism with signaling networks that drive tumor aggressiveness.

**Figure 1:**
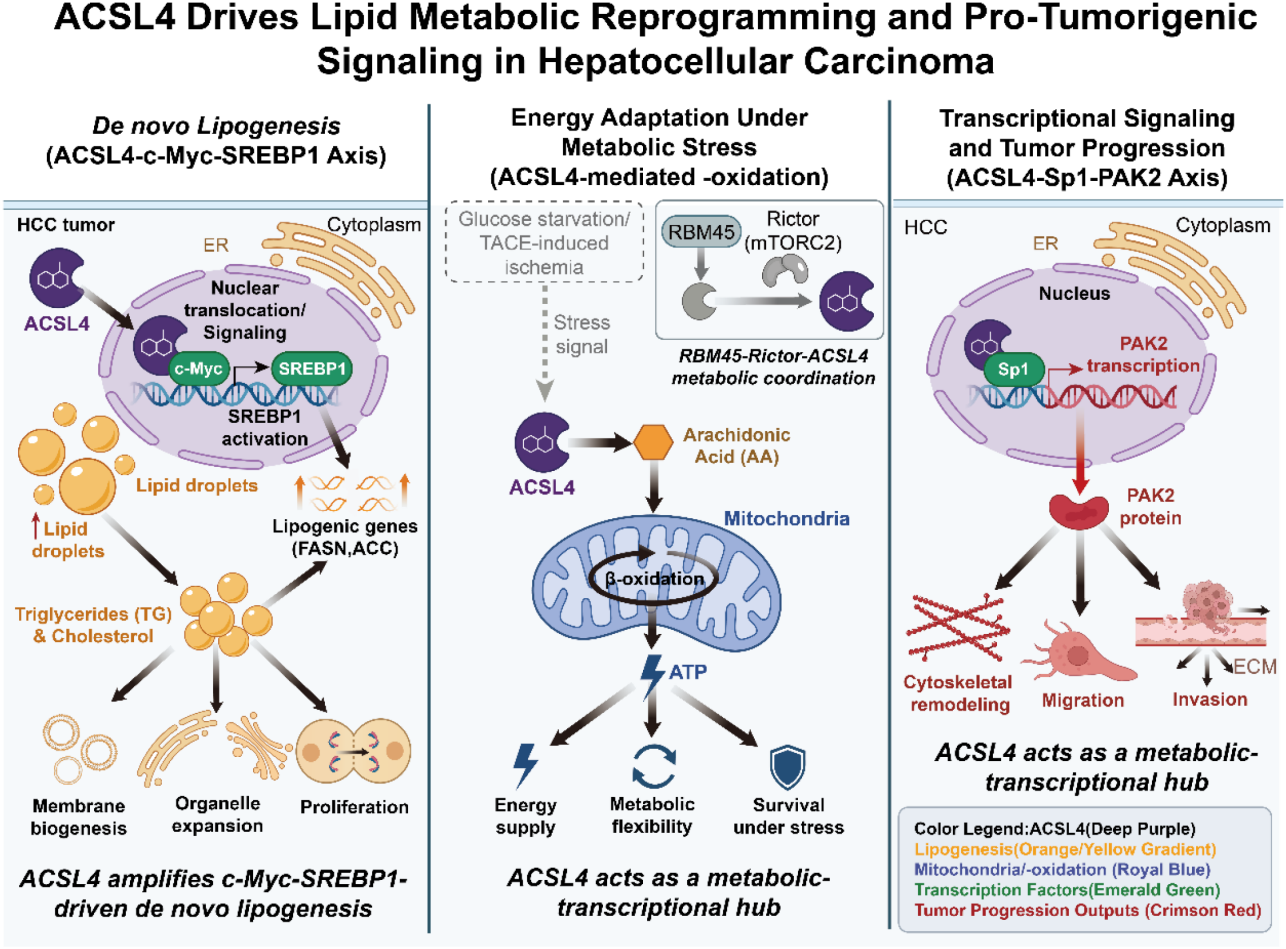
ACSL4 promotes hepatocellular carcinoma progression by integrating lipid anabolic signaling and metabolic stress adaptation. ACSL4 activates the c-Myc–SREBP1 axis to enhance *de novo* lipogenesis, while supporting arachidonic acid–driven β-oxidation under glucose deprivation or TACE-induced stress. Additionally, ACSL4 regulates Sp1-dependent PAK2 transcription to facilitate migration and invasion. Together, these pathways establish a pro-tumorigenic metabolic framework in HCC.

Collectively, these studies demonstrate that in metabolically permissive conditions, ACSL4 supports tumor growth by enhancing lipid synthesis, coordinating energy adaptation, and interfacing with oncogenic transcriptional programs. Through the integration of anabolic lipogenesis, stress-induced β-oxidation, and signaling network activation, ACSL4 establishes a pro-tumorigenic metabolic framework that enables HCC cells to proliferate, migrate, and survive under diverse environmental pressures (**Figure 1**).

## 3 ACSL4 as a determinant of ferroptotic sensitivity

While ACSL4 supports tumor growth through lipid metabolic remodeling under permissive conditions, its enzymatic activity simultaneously establishes a biochemical landscape that predisposes cells to ferroptotic death. This duality underscores the context-dependent nature of ACSL4 function in HCC. Ferroptosis sensitivity is critically dependent on the composition of membrane phospholipids, particularly the abundance of oxidizable PUFA-containing phospholipids (PUFA-PLs) (23, 24). As a central enzyme catalyzing PUFA activation, ACSL4 plays a decisive role in shaping this lipid substrate pool.

### 3.1 ACSL4 and PUFA incorporation into membrane phospholipids

Ferroptosis is initiated by the iron-dependent peroxidation of PUFA-PLs within cellular membranes. The susceptibility of tumor cells to ferroptosis is therefore determined not only by antioxidant defenses such as GPX4 and system Xc^-^ activity but also by the availability of peroxidation-prone lipid substrates. ACSL4 catalyzes the conversion of long-chain PUFAs—particularly arachidonic acid (AA) and adrenic acid—into acyl-CoA derivatives, enabling their incorporation into phosphatidylethanolamines and other membrane phospholipids (25). By expanding the pool of PUFA-PLs, ACSL4 effectively primes tumor cells for lipid peroxidation. Experimental depletion of ACSL4 markedly reduces PUFA incorporation into membranes and confers resistance to ferroptosis-inducing agents, whereas ACSL4 overexpression enhances lipid peroxidation and ferroptotic vulnerability. Thus, ACSL4 functions as a rate-limiting determinant of ferroptotic substrate availability, placing it upstream of redox imbalance and membrane damage. Importantly, ferroptosis sensitivity in HCC is highly heterogeneous, suggesting that ACSL4 expression levels and activity may define a ferroptosis-permissive metabolic state. In this sense, ACSL4 establishes the “ferroptotic readiness” of tumor cells, linking lipid remodeling directly to regulated cell death pathways.

### 3.2 Epigenetic regulation: the ETS1/miR-23a-3p/ACSL4 axis

The abundance of ACSL4—and consequently ferroptosis sensitivity—is dynamically regulated at the transcriptional and post-transcriptional levels. A notable example is the ETS1/miR-23a-3p/ACSL4 regulatory axis implicated in sorafenib resistance. In HCC models, the transcription factor ETS1 upregulates miR-23a-3p, which directly targets ACSL4 mRNA and suppresses its expression (26). Downregulation of ACSL4 reduces PUFA-PL accumulation, thereby lowering lipid peroxidation levels and attenuating ferroptotic cell death. Functionally, this axis contributes to acquired resistance to sorafenib, a multikinase inhibitor whose anti-tumor activity partially depends on the induction of ferroptotic stress. Inhibition of miR-23a-3p or restoration of ACSL4 expression re-sensitizes resistant HCC cells to sorafenib, highlighting the therapeutic relevance of ferroptosis modulation. These findings emphasize that ferroptotic susceptibility is not fixed but can be epigenetically tuned through microRNA-mediated control of ACSL4 expression. In this context, ACSL4 serves as a molecular gatekeeper linking non-coding RNA networks to redox-dependent cell death pathways.

### 3.3 Post-translational control: SIAH2-mediated degradation

Beyond transcriptional regulation, ACSL4 protein stability is subject to post-translational control, further adding a dynamic layer to ferroptosis regulation. The E3 ubiquitin ligase SIAH2 has been identified as a negative regulator of ACSL4 through ubiquitination-mediated proteasomal degradation. Elevated SIAH2 expression in HCC correlates with reduced ACSL4 levels and impaired ferroptotic responsiveness (27). Importantly, this regulatory mechanism extends beyond tumor cell–intrinsic metabolism and intersects with anti-tumor immunity. CD8^+^ T cells can promote tumor cell ferroptosis by disrupting cystine metabolism and increasing oxidative stress. However, SIAH2-driven degradation of ACSL4 diminishes the availability of peroxidizable lipid substrates, thereby limiting CD8^+^ T cell–mediated ferroptotic killing. Pharmacological inhibition of SIAH2 stabilizes ACSL4, enhances lipid peroxidation, and improves the efficacy of immune checkpoint blockade in preclinical models. These findings illustrate how post-translational modulation of ACSL4 can recalibrate tumor–immune interactions and influence immunotherapeutic outcomes.

**Figure 2:**
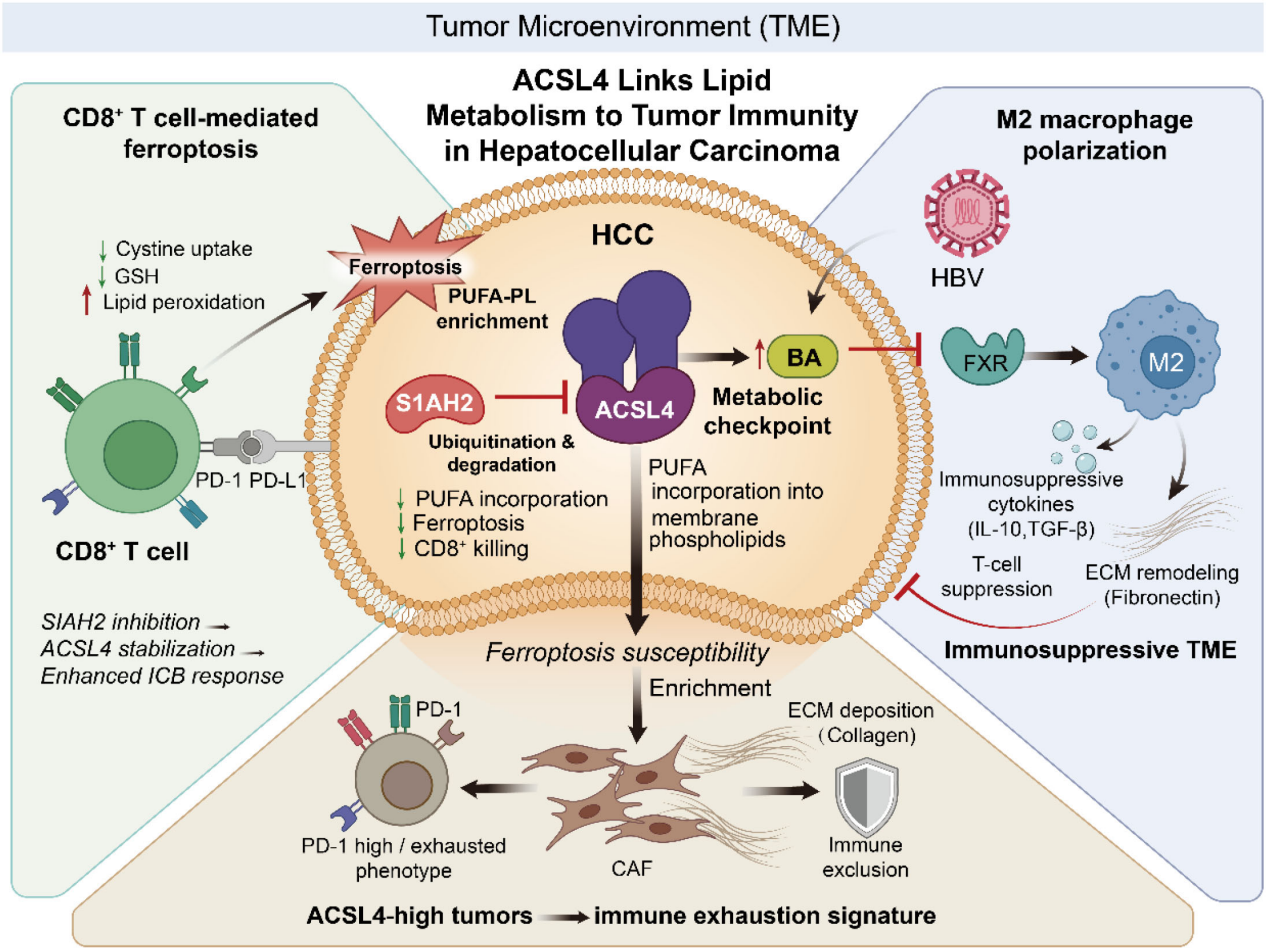
ACSL4 functions as an immunometabolic hub in hepatocellular carcinoma. By regulating PUFA incorporation and ferroptotic susceptibility, ACSL4 determines tumor sensitivity to CD8^+^ T cell–mediated killing. SIAH2-mediated degradation of ACSL4 promotes immune resistance, while bile acid–FXR dysregulation drives M2 macrophage polarization in HBV-associated HCC. ACSL4-high tumors are associated with CAF enrichment and immune exhaustion, collectively shaping an immunosuppressive microenvironment.

Collectively, these studies establish ACSL4 as a central determinant of ferroptotic sensitivity in HCC. By regulating PUFA incorporation into membrane phospholipids and being subject to multilayered transcriptional, epigenetic, and post-translational control, ACSL4 dynamically tunes the ferroptosis threshold of tumor cells. Ferroptosis sensitivity is therefore not static but continuously adjusted according to ACSL4 abundance, regulatory inputs, and therapeutic context. This dynamic regulation reinforces the concept of ACSL4 as a context-dependent metabolic switch—capable of promoting tumor growth under permissive conditions while simultaneously creating vulnerabilities that can be therapeutically exploited (**Figure 2**).

## 4 ACSL4 at the interface of tumor immunity

Beyond its tumor cell–intrinsic metabolic functions, ACSL4 increasingly emerges as a critical mediator linking lipid metabolism to anti-tumor immunity. The TME of HCC is characterized by complex immunometabolic interactions in which nutrient competition, lipid remodeling, and redox dynamics collectively shape immune cell function (28, 29). Through its role in regulating PUFA metabolism and ferroptotic susceptibility, ACSL4 acts as a metabolic checkpoint that influences both cytotoxic immune responses and immunosuppressive remodeling.

### 4.1 ACSL4 and CD8^+^ T-cell–mediated ferroptosis

CD8^+^ cytotoxic T lymphocytes (CTLs) exert anti-tumor effects not only through perforin–granzyme pathways but also by modulating tumor cell metabolism and redox balance (30, 31). Recent evidence indicates that CTLs can promote ferroptosis in tumor cells by disrupting cystine uptake, altering glutathione metabolism, and enhancing lipid peroxidation. Recent evidence indicates that CTLs can promote ferroptosis in tumor cells by disrupting cystine uptake, altering glutathione metabolism, and enhancing lipid peroxidation (32). This seminal study demonstrates that CD8^+^ T cell-derived IFNγ upregulates ACSL4 in tumor cells, directly linking immune signaling to ferroptotic execution, highlighting ACSL4 as a critical immune-metabolic interface. In this context, ACSL4 becomes a pivotal determinant of whether tumor cells remain susceptible to immune-mediated ferroptotic killing. The SIAH2–ACSL4 regulatory axis provides a mechanistic explanation for immune evasion (27). SIAH2-mediated ubiquitination and proteasomal degradation of ACSL4 reduce PUFA incorporation into membrane phospholipids, thereby lowering lipid peroxidation potential. Consequently, tumor cells with elevated SIAH2 expression exhibit reduced ferroptotic sensitivity and diminished responsiveness to CD8^+^ T-cell–mediated cytotoxicity. This mechanism represents a metabolic form of immune resistance. Importantly, pharmacological inhibition of SIAH2 stabilizes ACSL4 expression, enhances ferroptotic lipid accumulation, and potentiates the efficacy of immune checkpoint blockade (ICB) in preclinical models. These findings suggest that ACSL4 stabilization may synergize with PD-1/PD-L1–targeted therapies by increasing tumor vulnerability to T-cell–driven ferroptotic stress. Thus, ACSL4 serves as a molecular bridge between tumor lipid composition and immune effector function, positioning ferroptosis modulation as a promising strategy to overcome immunotherapy resistance.

### 4.2 ACSL4 and M2 macrophage polarization in HBV-associated HCC

In addition to shaping cytotoxic T-cell responses, ACSL4 influences the immunosuppressive landscape of the TME, particularly in the context of chronic hepatitis B virus (HBV) infection (33, 34). HBV-related HCC develops within an inflammatory and metabolically altered liver environment where bile acid (BA) signaling and nuclear receptor pathways are dysregulated. Recent studies demonstrate that ACSL4 modulates bile acid metabolism and farnesoid X receptor (FXR) signaling, thereby contributing to macrophage polarization. Elevated ACSL4 expression correlates with increased BA accumulation and reduced FXR activity, conditions that favor the polarization of tumor-associated macrophages (TAMs) toward an M2-like phenotype. M2 macrophages promote tumor progression through immunosuppressive cytokine production, extracellular matrix remodeling, and suppression of cytotoxic T-cell activity. Conversely, ACSL4 silencing restores FXR signaling, reduces bile acid accumulation, and attenuates M2 polarization, suggesting that ACSL4-driven lipid remodeling indirectly orchestrates immune suppression. These findings highlight a previously underappreciated connection between fatty acid activation, bile acid homeostasis, and macrophage immunobiology. In HBV-associated HCC, ACSL4 therefore contributes to the establishment of a tolerogenic microenvironment that supports tumor persistence.

### 4.3 Association with cancer-associated fibroblasts and exhausted immune microenvironment

Spatial transcriptomic analyses and clinical correlation studies further support a broader immunomodulatory role for ACSL4. In HCC patient cohorts, ACSL4 expression has been associated with increased infiltration of cancer-associated fibroblasts (CAFs) and features of an exhausted immune microenvironment. CAFs contribute to extracellular matrix deposition, metabolic reprogramming, and immune exclusion. The positive spatial correlation between ACSL4 and CAF-enriched niches suggests that lipid metabolic remodeling may co-evolve with stromal activation. Moreover, tumors with high ACSL4 expression often display transcriptional signatures indicative of immune exhaustion, including upregulation of inhibitory checkpoint molecules and reduced cytotoxic effector gene expression. Clinically, ACSL4 positivity correlates with poorer prognosis and more aggressive tumor characteristics, reinforcing its association with an immunosuppressive TME. Although causality remains to be fully elucidated, these data imply that ACSL4-driven metabolic states may foster stromal–immune crosstalk that dampens effective anti-tumor immunity.

Collectively, emerging evidence positions ACSL4 at the nexus of tumor lipid metabolism and immune regulation. By modulating ferroptotic susceptibility, macrophage polarization, bile acid signaling, and stromal interactions, ACSL4 bridges tumor cell metabolic remodeling with immune cell function within the TME. Through this immunometabolic integration, ACSL4 not only influences tumor growth and survival but also shapes the responsiveness of HCC to immunotherapeutic interventions.

## 5 Context-dependent dual roles: a metabolic switch model

The seemingly paradoxical roles of ACSL4 in hepatocellular carcinoma—promoting tumor growth on one hand while enhancing ferroptotic vulnerability on the other—cannot be reconciled by a unidirectional oncogenic model. Instead, accumulating evidence supports a context-dependent metabolic switch paradigm, in which ACSL4 dynamically redirects lipid flux toward distinct biological outputs depending on environmental cues and therapeutic pressures. Rather than functioning as a static oncogene or tumor suppressor, ACSL4 acts as a metabolic rheostat that integrates substrate availability, transcriptional regulation, and immune signaling to determine cellular fate.

### 5.1 Growth-promoting mode: lipid anabolism and metabolic adaptation

In metabolically permissive environments—characterized by sufficient nutrient availability and limited oxidative stress—ACSL4 primarily supports tumor progression. Through activation of long-chain PUFAs and amplification of the c-Myc–SREBP1 lipogenic axis, ACSL4 enhances *de novo* lipid synthesis, leading to triglyceride accumulation, membrane expansion, and signaling platform formation. This anabolic lipid remodeling facilitates rapid proliferation and metastatic potential. Simultaneously, ACSL4 contributes to metabolic flexibility by enabling the utilization of fatty acids as alternative energy substrates. Under conditions such as glucose deprivation or ischemia induced by TACE, ACSL4-mediated activation of arachidonic acid fuels mitochondrial β-oxidation, sustaining ATP production and promoting survival. In this growth-promoting mode, ACSL4 enhances both anabolic and catabolic lipid pathways, providing tumor cells with adaptive resilience. Importantly, in this state, antioxidant systems and redox buffering mechanisms may counterbalance lipid peroxidation, preventing ferroptotic execution despite increased PUFA flux. Thus, lipid remodeling favors tumor expansion rather than cell death.

### 5.2 Ferroptosis-sensitizing mode: lipid peroxidation vulnerability

Under conditions of elevated oxidative stress, therapeutic intervention, or immune activation, the same ACSL4-driven PUFA enrichment can shift cellular outcomes toward ferroptosis. By increasing the incorporation of peroxidation-prone PUFAs into membrane phospholipids, ACSL4 expands the substrate pool required for iron-dependent lipid peroxidation. In the context of sorafenib treatment, higher ACSL4 levels correlate with enhanced ferroptotic sensitivity, whereas suppression via the ETS1/miR-23a-3p axis reduces lipid peroxidation and confers drug resistance. Similarly, during CD8^+^ T-cell–mediated immune responses, ACSL4 abundance determines whether tumor cells can undergo ferroptotic death upon redox disruption (26). Stabilization of ACSL4 enhances immune-mediated tumor clearance and may improve responses to immune checkpoint blockade. Thus, in this ferroptosis-sensitizing mode, ACSL4 transforms from a facilitator of lipid-driven growth into a liability that renders tumor cells vulnerable to oxidative collapse. The biochemical substrate pool remains similar, but the cellular redox context determines whether lipid remodeling supports survival or triggers death.

### 5.3 Determinants of switching: what controls ACSL4 output?

The transition between growth-promoting and ferroptosis-sensitizing modes is not arbitrary but is governed by a multilayered regulatory architecture that integrates therapeutic pressure, microenvironmental cues, molecular regulation, and metabolic substrate dynamics. ACSL4 does not intrinsically dictate a singular biological outcome; rather, its functional output is continuously shaped by the contextual landscape in which tumor cells reside.

Therapeutic interventions represent one of the most decisive modulators of ACSL4 activity. Distinct treatments impose fundamentally different metabolic stresses that redirect lipid flux. TACE, for example, induces ischemia and nutrient deprivation, conditions that favor fatty acid oxidation–dependent energy adaptation. In this setting, ACSL4-mediated activation of arachidonic acid supports mitochondrial ATP production and enhances tumor survival. In contrast, targeted therapies such as sorafenib, as well as immune checkpoint blockade, elevate oxidative stress and perturb redox homeostasis. Under these circumstances, ACSL4-driven enrichment of PUFA-containing phospholipids amplifies lipid peroxidation, thereby shifting the balance toward ferroptotic vulnerability. Thus, ACSL4 output is intrinsically therapy-dependent. Beyond therapeutic context, microenvironmental conditions critically determine whether ACSL4-fueled lipid remodeling sustains survival or precipitates death. Hypoxia, glucose deprivation, inflammatory cytokine signaling, and iron availability collectively shape cellular redox balance and membrane susceptibility to oxidation. In hypoxic yet antioxidant-competent environments, enhanced lipid synthesis and β-oxidation may confer adaptive advantages without triggering ferroptosis. Conversely, in iron-rich or inflammatory niches characterized by heightened oxidative stress, the same PUFA-enriched membranes become liabilities, predisposing cells to peroxidative collapse. The redox tone of the tumor microenvironment therefore acts as a functional filter through which ACSL4 activity is interpreted.

At the molecular level, ACSL4 abundance is dynamically regulated through post-transcriptional and post-translational mechanisms that fine-tune ferroptotic thresholds. MicroRNAs, including miR-23a-3p and miR-145-5p, modulate ACSL4 mRNA stability and translation, while E3 ubiquitin ligases such as SIAH2 control protein turnover via proteasomal degradation. These regulatory circuits allow rapid recalibration of ACSL4 expression in response to stress signals, thereby adjusting the pool of peroxidizable phospholipids and altering susceptibility to ferroptosis. Such multilayered regulation underscores that ferroptotic readiness is an actively maintained state rather than a fixed trait. Equally important is the availability and routing of metabolic substrates. The ultimate fate of activated PUFAs depends on the balance between membrane incorporation, lipid droplet sequestration, β-oxidation, and antioxidant defense systems such as GPX4. When PUFA flux is directed toward membrane phospholipid remodeling in the presence of compromised redox buffering, lipid peroxidation is favored. However, if PUFAs are efficiently stored in lipid droplets or metabolized through controlled oxidative pathways while antioxidant capacity remains intact, ferroptosis may be avoided despite high ACSL4 activity. In this regard, substrate flux directionality—rather than enzyme abundance alone—determines cellular outcome.

Taken together, these converging influences form a multilayered regulatory network that dictates ACSL4 functional output. The enzyme operates at the nexus of therapy-induced stress, microenvironmental redox status, regulatory RNA networks, ubiquitin-mediated turnover, and lipid flux partitioning. It is this integrative positioning that enables ACSL4 to function as a context-dependent metabolic switch rather than a binary oncogenic or tumor-suppressive factor.

## 6 Therapeutic implications

The recognition of ACSL4 as a context-dependent metabolic switch provides a strategic framework for therapeutic intervention. Rather than viewing ACSL4 solely as a target to inhibit or activate, therapeutic approaches should aim to manipulate the metabolic and redox context in which ACSL4 operates, thereby directing lipid remodeling toward ferroptotic vulnerability rather than tumor adaptation. This perspective opens multiple avenues for combination therapy, regulatory targeting, and biomarker-driven patient stratification.

### 6.1 Ferroptosis-based combination strategies

Given its central role in determining ferroptotic substrate availability, ACSL4 represents a critical node for ferroptosis-based therapeutic enhancement. In sorafenib-treated HCC, induction of ferroptotic stress contributes substantially to anti-tumor efficacy. However, resistance frequently emerges through suppression of ACSL4 expression, often mediated by the ETS1/miR-23a-3p axis. Restoration of ACSL4 expression or inhibition of miR-23a-3p re-sensitizes tumor cells to sorafenib by reinstating PUFA incorporation and lipid peroxidation capacity. These findings support rational combination strategies that simultaneously induce oxidative stress and preserve or augment ACSL4-driven PUFA flux. Sorafenib combined with ferroptosis inducers—such as system Xc^-^ inhibitors, GPX4 inhibitors, or iron-modulating agents—may push ACSL4-enriched membranes beyond the oxidative threshold required for ferroptotic collapse. Importantly, patient tumors with higher baseline ACSL4 expression may be particularly susceptible to such strategies, suggesting a biomarker-guided approach. Furthermore, in the immunotherapy setting, ACSL4 stabilization may enhance CD8^+^ T-cell–mediated ferroptotic killing. Thus, integrating ferroptosis-inducing agents with immune checkpoint blockade could represent a synergistic therapeutic paradigm, especially in metabolically primed tumors.

### 6.2 Targeting the ACSL4 regulatory network

Because ACSL4 activity is dynamically regulated, targeting its upstream modulators offers additional therapeutic leverage. Post-transcriptional regulators such as miR-23a-3p and miR-145-5p represent potential intervention points. Inhibiting miR-23a-3p may restore ACSL4 expression and overcome sorafenib resistance, while reconstitution of miR-145-5p—often downregulated in HCC—may suppress tumor progression through controlled modulation of ACSL4 and associated oncogenic pathways. At the post-translational level, inhibition of SIAH2-mediated ubiquitination provides a promising strategy to stabilize ACSL4 protein. By preventing proteasomal degradation, SIAH2 inhibitors may increase tumor ferroptotic susceptibility and enhance responsiveness to immune checkpoint blockade. Importantly, such approaches do not directly target ACSL4 enzymatic activity but instead modulate its abundance and context-dependent function, potentially allowing for more nuanced therapeutic control. These regulatory-layer interventions highlight that ACSL4-centered therapy does not necessarily require enzymatic inhibition. Instead, modulating the regulatory circuitry surrounding ACSL4 may shift the metabolic balance toward tumor vulnerability.

### 6.3 Precision medicine and biomarker potential

The dual nature of ACSL4 necessitates a precision medicine approach. Rather than uniformly suppressing ACSL4, patient stratification based on metabolic context and therapeutic modality may be required. In the setting of TACE, where ischemic and nutrient stress predominate, elevated ACSL4 expression may confer metabolic adaptability and resistance. In this context, ACSL4 inhibition could potentially impair fatty acid-dependent energy compensation and improve therapeutic outcomes. Conversely, in patients receiving sorafenib or immunotherapy, higher ACSL4 levels may predict enhanced ferroptotic responsiveness and improved treatment efficacy. Supporting this concept, Feng et al. identified ACSL4 as a predictive biomarker of sorafenib sensitivity in hepatocellular carcinoma, indicating that ACSL4-based stratification may help identify patients more likely to benefit from ferroptosis-related therapeutic vulnerability (35).

Immunohistochemical assessment of ACSL4 expression in tumor tissues may therefore serve as a practical stratification tool. In addition, recent clinicopathological and spatial transcriptomic analyses showed that ACSL4 expression is associated with prognosis, cancer-associated fibroblast enrichment, and anti-tumor immunity in HCC, further supporting its biomarker potential at the tumor ecosystem level (36). Integration of ACSL4 status with ferroptosis-related gene signatures, immune infiltration markers, and metabolic profiling could refine prognostic models and guide treatment selection. Although prospective clinical validation specifically centered on ACSL4 remains limited, ongoing biomarker-oriented clinical studies in advanced HCC highlight the growing translational relevance of molecular stratification. For example, an observational study is evaluating predictive biomarkers in patients with advanced HCC treated with atezolizumab plus bevacizumab, and another study is assessing predictive biomarkers in patients receiving systemic therapy for advanced HCC (37, 38). These ongoing efforts provide a clinical framework into which ACSL4 may be incorporated in future biomarker-guided precision treatment strategies.

## 7 Future perspectives

Although significant progress has been made in delineating the dual roles of ACSL4 in hepatocellular carcinoma, several critical questions remain unresolved. Addressing these gaps will be essential for translating the metabolic switch model of ACSL4 into clinically actionable strategies. One important area of investigation concerns the potential existence of isoform-specific or context-specific functional differences of ACSL4. While ACSL4 is generally studied as a single enzymatic entity, alternative splicing, post-translational modifications, or differential subcellular localization may confer distinct biological outputs. It remains unclear whether ACSL4 activity in the endoplasmic reticulum, mitochondria-associated membranes, or lipid droplets produces divergent effects on ferroptosis sensitivity and metabolic adaptation. Dissecting spatially resolved ACSL4 functions could refine our understanding of how lipid flux is partitioned between anabolic growth and oxidative vulnerability.

Another unresolved question is the degree of intratumoral heterogeneity in ACSL4 expression and activity. Single-cell transcriptomic and spatial multi-omics technologies may reveal distinct ACSL4-high and ACSL4-low tumor subpopulations coexisting within the same lesion. Such heterogeneity could explain variable therapeutic responses to ferroptosis-inducing agents or immunotherapy. Furthermore, differential ACSL4 expression across tumor cells, cancer-associated fibroblasts, macrophages, and endothelial cells may indicate cell type–specific metabolic dependencies that are currently underexplored. Emerging evidence also suggests potential crosstalk between lipid metabolism and epitranscriptomic regulation (39–41). Whether ACSL4 expression or activity is modulated by m6A-dependent RNA methylation, or whether ACSL4-driven lipid remodeling influences RNA modification landscapes, remains an open question. Given the central role of m6A in regulating metabolic gene networks and therapeutic resistance in HCC, integrating ACSL4 into the broader framework of epigenetic–metabolic coupling may uncover additional regulatory layers.

In parallel, the relationship between ACSL4 and lipid droplet biogenesis warrants deeper investigation. Lipid droplets serve as dynamic reservoirs that buffer excess fatty acids and protect cells from lipotoxicity. It is conceivable that ACSL4-driven PUFA activation is temporally directed toward either membrane phospholipid incorporation or lipid droplet sequestration depending on cellular redox status (42–44). Understanding how membrane dynamics, phospholipid remodeling, and lipid storage intersect with ACSL4 activity could clarify the mechanistic threshold between metabolic adaptation and ferroptotic execution. Finally, the long-term metabolic evolution of the cirrhotic liver microenvironment may profoundly influence ACSL4 function. Most HCC cases arise in the setting of chronic liver disease characterized by fibrosis, inflammation, and altered bile acid metabolism (45, 46). Persistent oxidative stress, iron accumulation, and cytokine exposure may precondition tumor cells toward specific ACSL4-dependent states. Longitudinal studies examining ACSL4 dynamics during cirrhosis-to-carcinoma progression could provide insights into how chronic metabolic remodeling shapes ferroptotic vulnerability and immune responsiveness.

Collectively, future research should move beyond viewing ACSL4 as a static metabolic enzyme and instead investigate its spatial regulation, cellular heterogeneity, epigenetic coupling, and long-term microenvironmental interactions. Such integrative approaches will be essential to fully exploit ACSL4-driven lipid remodeling as a therapeutic vulnerability. Understanding ACSL4 as a context-dependent metabolic switch may enable rational design of ferroptosis-enhancing and immunometabolic therapeutic strategies in HCC.

## 8 Conclusion

ACSL4 occupies a unique and paradoxical position in hepatocellular carcinoma biology. As a central regulator of polyunsaturated fatty acid activation, it simultaneously fuels lipid-driven tumor growth and establishes the biochemical foundation for ferroptotic vulnerability. This duality cannot be adequately explained by traditional oncogene-centric paradigms. Instead, emerging evidence supports a context-dependent metabolic switch model, in which ACSL4 dynamically integrates lipid remodeling, redox balance, immune pressure, and therapeutic stress to determine cellular fate. Under metabolically permissive conditions, ACSL4 amplifies lipogenic signaling, enhances fatty acid oxidation–mediated energy flexibility, and cooperates with oncogenic transcriptional networks to promote tumor progression. Conversely, in oxidative or immune-activated contexts, ACSL4-driven enrichment of PUFA-containing phospholipids sensitizes tumor cells to ferroptosis and potentiates anti-tumor immunity. The functional outcome of ACSL4 activity is therefore dictated not by its intrinsic enzymatic function alone, but by the surrounding metabolic and microenvironmental landscape.

Importantly, ACSL4 regulation is multilayered and dynamically modulated through transcriptional programs, microRNA networks, ubiquitin-mediated degradation, and substrate flux partitioning. This regulatory complexity provides multiple intervention points for therapeutic manipulation. Rather than uniformly suppressing ACSL4, future strategies may aim to redirect its metabolic output—leveraging lipid remodeling to enhance ferroptosis and immunotherapeutic efficacy while limiting adaptive energy support. In this light, ACSL4 represents more than a metabolic enzyme; it is a nodal integrator of tumor metabolism, ferroptotic sensitivity, and immune responsiveness in HCC. Conceptualizing ACSL4 as a context-dependent metabolic switch offers a unifying framework to reconcile its dual roles and provides a rational basis for designing next-generation ferroptosis-enhancing and immunometabolic therapeutic strategies.
